# PTX3 gene activation in EGF-induced head and neck cancer cell metastasis

**DOI:** 10.18632/oncotarget.3482

**Published:** 2015-03-08

**Authors:** Wei-Chiao Chang, Shuo-Lun Wu, Wan-Chen Huang, Jinn-Yuan Hsu, Shih-Hung Chan, Ju-Ming Wang, Jhih-Peng Tsai, Ben-Kuen Chen

**Affiliations:** ^1^ Department of Clinical Pharmacy, Master Program for Clinical Pharmacogenomics and Pharmacoproteomics, School of Pharmacy, Taipei Medical University, Taipei, Taiwan, ROC; ^2^ Institute of Bioinformatics and Biosignal Transduction, College of Bioscience and Biotechnology, National Cheng Kung University, Tainan, Taiwan, ROC; ^3^ Institute of Basic Medical Sciences, College of Medicine, National Cheng Kung University, Tainan, Taiwan, ROC; ^4^ Department of Internal Medicine, National Cheng Kung University Hospital, College of Medicine, National Cheng Kung University, Tainan, Taiwan, ROC; ^5^ Department of Pharmacology, College of Medicine, National Cheng Kung University, Tainan, Taiwan, ROC; ^6^ Graduate Institute of Clinical Medicine, College of Medicine, Kaohsiung Medical University, Kaohsiung, Taiwan, ROC; ^7^ Department of Pharmacy, Taipei Medical University-Wanfang Hospital, Taipei, Taiwan, ROC; ^8^ Center for Biomarkers and Biotech Drugs, Kaohsiung Medical University, Kaohsiung, Taiwan, ROC; ^9^ Institute for Cancer Biology and Drug Discovery, College of Medical Science and Technology, Taipei Medical University, Taipei, Taiwan, ROC

**Keywords:** PTX3, EGF, cancer metastasis

## Abstract

Overexpression of the epidermal growth factor (EGF) receptor (EGFR) is associated with enhanced invasion and metastasis in head and neck squamous cell carcinoma (HNSCC). Long Pentraxin PTX3 is involved in immune escape in cancer cells. Here, we identified PTX3 as a promoting factor that mediates EGF-induced HNSCC metastasis. EGF-induced PTX3 transcriptional activation is via the binding of c-Jun to the activator protein (AP)-1 binding site of the PTX3 promoter. PI3K/Akt and NF-κB were essential for the PTX3 activation. EGF-induced PTX3 expression was blocked in c-Jun- and NF-κB-knockdown cells. EGF-mediated PTX3 secretion resulted in the enhancement of cell migration and invasion, and interactions between cancer and endothelial cells. The tail-vein injection animal model revealed that depletion of PTX3 decreased EGF-primed tumor cell metastatic seeding of the lungs. In addition, fibronectin, matrix metalloproteinase-9 (MMP9) and E-cadherin were essential components in EGFR/PTX3-mediated cancer metastasis. In conclusion, PI3K/Akt and NF-κB-dependent regulation of AP-1 mediates PTX3 transcriptional responses to EGF. Autocrine production of EGF-induced PTX3 in turn induces metastatic molecules, activating inflammatory cascades and metastasis.

## INTRODUCTION

Head and neck cancers are one of the most common human cancers, with an annual incidence of >500,000 cases worldwide [1]. More than 90% of head and neck cancers are of squamous cell histology and originate in the lip, tongue, gum, oropharynx, nasopharynx, oral cavity, and hypopharynx [2]. The main risk factors in head and neck squamous cell carcinoma (HNSCC) include the use of tobacco and alcohol and human papillomavirus (HPV) infection [3]. Several tumor markers are overexpressed in HNSCC. Specifically, expressions of epidermal growth factor (EGF) receptor (EGFR) and its ligand, transforming growth factor (TGF)-α, are frequently observed in the carcinogenesis of HNSCC [4, 5].

PTX3 is the prototypic long pentraxin produced by resident or innate immune cells in peripheral tissues in response to inflammatory signals and toll-like receptor (TLR) activation [6]. The physiological roles of PTX3 in innate immunity and inflammation have been reported [6, 7]. PTX3 binds specific pathogens, such as fungi, bacteria, and viruses to promote phagocytosis and consequent clearance of the pathogen [8-10]. For example, the interaction of PTX3 with C1q results in the activation of classic complement cascade, when C1q is plastic-immobilized. However, when an interaction occurs in the fluid phase, inhibition of C1q hemolytic activity is observed, suggesting a possible inhibitory effect by competitive blocking of relevant sites [11]. High expression of PTX3 was noted in diverse infectious disorders including sepsis, tuberculosis, dengue infection, and autoimmune disorders [7]. The findings suggest a potential role of PTX3 in infectious diseases.

PTX3 can be regulated by several stimuli, such as proinflammatory cytokines (interleukin (IL)-1β, tumor necrosis factor (TNF)-α), TLR agonists (lipopolysaccharides (LPSs)), distinct microbial moieties, and microorganisms [12]. In lung epithelial cells, TNF-α mediated PTX3 activation is via c-Jun N-terminal kinase (JNK) pathways [13]. In addition, the transcription factor CCAAT/enhancer binding protein delta (CEBPD) is a key molecule that enhances PTX3 expression in astrocytes [14]. EGFR expression has been reported to positively correlate with the levels of PTX3 in prostate tissues [15]. The results implied that activation of growth factor signaling may regulate PTX3 expression. Indeed, increasing evidence suggests that PTX3 expression may be a new diagnostic and prognostic marker of cancers, including pancreatic carcinoma, glioma malignancy, and lung carcinoma [16-18], however, neither the mechanisms of PTX3 transcriptional activation nor the functional role of PTX3 in the EGF-mediated HNSCC metastasis has clearly described. Herein, we demonstrated a mechanistic study on the transcriptional activation of PTX3. Activation of PI3k/Akt and NFκB pathways and binding of AP1 site of the PTX3 promoter are essential for EGF-induced PTX3 activation. Importantly, tail-vein injection animal model revealed that depletion of PTX3 significantly blocked EGF-primed tumor cell metastatic seeding of the lungs. In short, our results identified a functional role for PTX-3, namely the activation of a downstream signaling resulting in metastatic molecules fibronectin and MMP9 expression.

## RESULTS

### EGF regulates PTX3 expression in head and neck cancer cell lines

**Figure 1:**
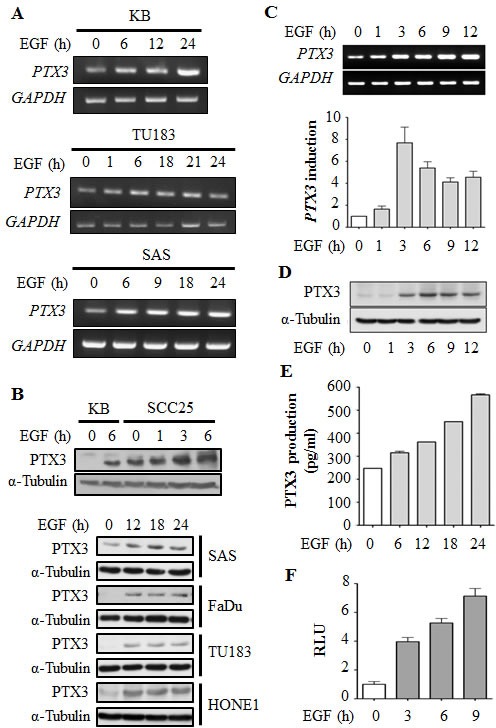
EGF induces transcriptional activation of PTX3 gene expression in head and neck squamous cell carcinoma (HNSCC) cell lines (A) HNSCC cell lines were treated with 50 ng/ml EGF for a period of time as indicated. Expressions of *PTX3* and *GAPDH* mRNA were analyzed by an RT-PCR and examination in 2% agarose gels. (B) Lysates of cells were prepared and subjected to SDS-PAGE and analyzed by Western blotting with antibodies against PTX3 and α-tubulin. (C) KB cells were treated with 50 ng/ml EGF for a period of time as indicated. Expressions of *PTX3* and *GAPDH* mRNA were analyzed by an RT-PCR (upper panel) and a real-time quantitative PCR (lower panel). Relative levels of *PTX3* were normalized to *GADPH*. Values represent the mean ± S.E. of three independent experiments. (D) Lysates of EGF-treated KB cells were prepared and subjected to SDS-PAGE and analyzed by Western blotting with antibodies against PTX3 and α-tubulin. (E) KB cells were treated with 50 ng/ml EGF for a period of time, and then conditioned medium was collected to analyze PTX3 protein by an ELISA. Values represent the mean ± S.E. of three independent experiments. (F) KB cells were transfected with 0.5 μg PTX3 promoter construct by lipofection and then treated with 50 ng/ml EGF for various times as indicated. Luciferase activities and protein concentrations were then determined and normalized. Values represent the mean ± S.E. of three determinations.

To investigate the association of the PTX3 gene expression signature with HNSCC, data mining on the cancer microarray database, Oncomine 4.0 (Oncomine DB at http://www.oncomine.org) [19], was used. PTX3 expressions between normal and malignant or metastatic tissues from HNSCC patients were analyzed using published datasets. The expression level of PTX3 in malignant tissues was higher than that in normal tissues in HNSCC patients (Supplementary Fig. 1A). Significantly, PTX3 expression was obvious in metastatic tissues (Supplementary Fig. 1B). These results suggested that PTX3 mRNA was overexpressed (*p* < 0.05) in clinical HNSCC tissues. We further studied PTX3 expression in various malignant tumor cells treated with EGF. Interestingly, we found that EGF significantly induced PTX3 gene expression (Fig. 1A) and protein production (Fig. 1B) in time-dependent manners in head and neck cancer cell lines, but a tiny induction was observed in HeLa cells (Supplementary Fig. 2). The RT-PCR and real-time quantitative RT-PCR results showed that the PTX3 mRNA level was substantially elevated and reached a peak after 3 h of EGF treatment (Fig. 1C). These results revealed that PTX3 was significantly induced by EGF in head and neck cancer cells. To further confirm the induction of PTX3 by EGF, the expression and secretion of PTX3 were examined in cell lysates and conditioned media, respectively. As shown in Fig. 1D and 1E, EGF also increased PTX3 protein production and secretion in cultured media in time-dependent manners. To investigate whether the alteration of transcriptional activity was responsible for EGF-induced PTX3 gene expression, we studied the effects of EGF on PTX3 promoter activity using a luciferase reporter assay. As shown in Fig. 1F, EGF induced substantial PTX3 promoter activity in a time-dependent manner. These results revealed that EGF stimulated PTX3 expression through transcriptional activation, resulting in the generation of PTX3.

### The EGF induces PTX3 expression through PI3K/Akt- and NF-κB-dependent pathways

**Figure 2:**
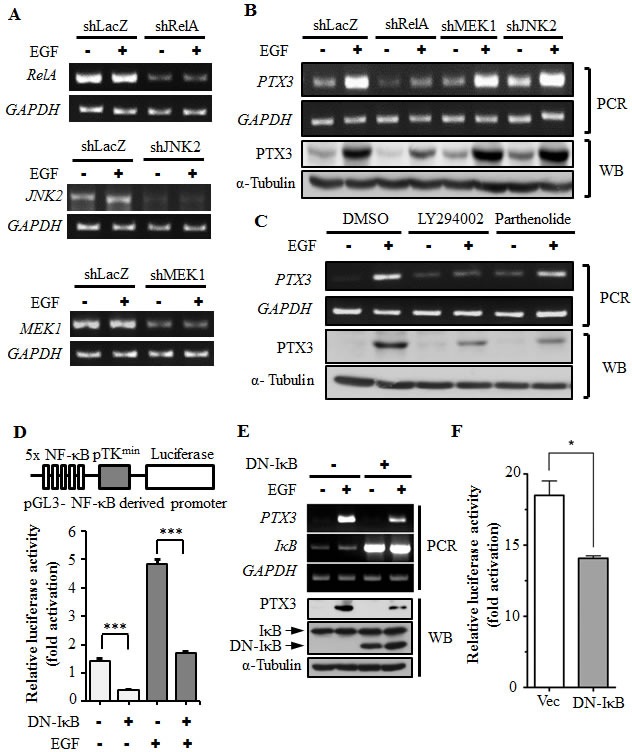
Activation of NF-κB is essential for EGF-induced PTX3 expression (A) RelA-, MEK1-, and JNK2-deficient cell lines were selected by infecting KB cells with a lentivirus containing an expression vector encoding short hairpin (sh)RNA against RelA (shRelA), MEK1 (shMEK1), and JNK2 (shJNK2). Expressions of *RelA, MEK1, JNK2*, and *GAPDH* mRNAs were analyzed by an RT-PCR and examined in 2% agarose gels. shLacZ, negative control. (B) shRNA containing cells was treated with 50 ng/ml EGF for 3 h, and expressions of PTX3 mRNA and protein were respectively analyzed by an RT-PCR and Western blotting (WB). shLacZ, negative control. (C) KB cells were treated with 25 μM LY294002, 10 μM parthenolide, or 0.1% DMSO for 1 h, followed by treatment with 50 ng/ml EGF for 3 h. Expressions of PTX3 mRNA and protein were respectively analyzed by an RT-PCR and WB. (D) The construct of the pTK promoter with five repeated NF-κB-binding sites bearing the luciferase gene is presented (upper panel). KB cells were transfected with 0.5 μg pTK-NF-κB promoter, 1 μg dominant negative IκB (DN-IκB) expression vector, and 1 μg control vector by lipofection and then treated with 50 ng/ml EGF for 6 h. Luciferase activities and protein concentrations were then determined and normalized (lower panel). (E) KB cells were transfected with 1 μg DN-IκB expression vector or 1 μg control vector by lipofection and then treated with 50 ng/ml EGF for 6 h before extraction of RNA or lysates. Expressions of PTX3, IκB, GAPDH, and α-tubulin mRNAs and proteins were respectively analyzed by an RT-PCR (PCR) and Western blotting (WB). (F) KB cells were transfected with 0.5 μg PTX3 promoter construct, 1 μg DN-IκB expression vector, or 1 μg control vector by lipofection and then treated with 50 ng/ml EGF for 6 h. Luciferase activities and protein concentrations were then determined and normalized. Values represent the mean ± S.E. of three determinations.

To clarify the signaling pathways involved in the regulation of EGF-induced PTX3 expression, downstream targets regulated by EGF were examined. As shown in Supplementary Fig. 3A, EGF stimulated activation of ERK1/2 and Akt by enhancing kinase phosphorylation in head and neck cancer cells. In addition, EGF also increased the phosphorylation IκBα, resulting in a decrease in the IκBα level (Supplementary Fig. 3A). Subsequently, we investigated whether the decrease in IκBα led to an increase in the nuclear translocation of NF-κB in EGF-treated cells. Indeed, EGF significantly induced the nuclear translocation of NF-κB (Supplementary Fig. 3B). These results suggest that EGF activates the ERK and Akt/NF-κB signaling pathways in HNSCC. To clarify which EGF-activated signaling pathways are involved in regulating PTX3 expression, stable cell lines with RelA-, MEK1-, and JNK2-knockdown via short hairpin (sh)RNA knockdown of RelA (shRelA), MEK1 (shMEK1), and JNK2 (shJNK2), respectively, were confirmed and used (Fig. 2A). As shown in Fig. 2B, EGF-induced expression of PTX3 was inhibited in shRelA cells, but not in shMEK1 or shJNK2 cells. In addition, LY294002 and parthenolide, inhibitors of phosphoinositide 3-kinases (PI3Ks) and NF-κB, respectively inhibited EGF-induced PTX3 mRNA and protein expressions (Fig. 2C). To further study the effect of permanently preventing NF-κB activation on EGF-induced PTX3 expression, we utilized a dominant negative form of IκB (DN-IκB) that lacked all N-terminal phosphorylation sites, thus it is resistant to degradation but still had the ability to bind to NF-κB [20, 21]. We found that the overexpression of DN-IκB inhibited the basal activity of NF-κB, and also significantly reduced EGF-induced NF-κB activity (Fig. 2D). Consistently, EGF-induced PTX3 mRNA and protein expressions were dramatically reduced in DN-IκB-expressing cells (Fig. 2E). In addition, DN-IκB also inhibited EGF-induced PTX3 promoter activity (Fig. 2F). These results indicated that EGF-induced PTX3 expression was, at least in part, through activation of the PI3K/Akt and NF-κB pathways.

### EGF induces the binding of c-Jun to AP1 sites on the PTX3 promoter

**Figure 3:**
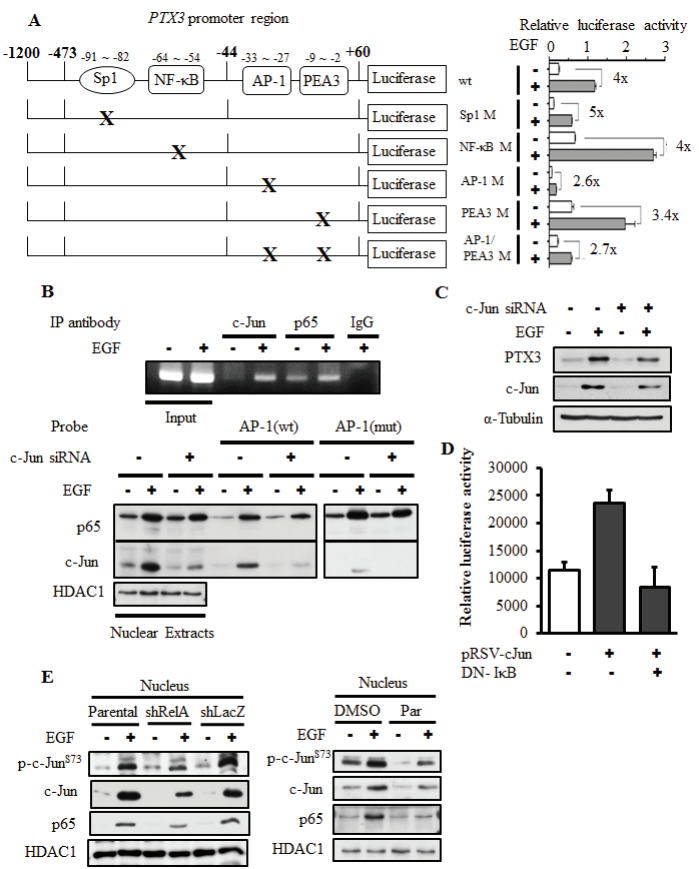
The binding of c-Jun to AP1-binding site located on the PTX3 promoter is essential for EGF-induced promoter activity (A) Schematic representation of reporter constructs with mutations of transcriptional factor-binding sites shown on the left. Luciferase activities of these reporter constructs in KB cells treated with 50 ng/ml EGF for 6 h are shown on the right. Values of luciferase activities are presented as the mean ± S.E. of three determinations. (B) KB cells were transfected with or without 20 nM c-Jun siRNA oligonucleotides by lipofection. After 50 ng/ml EGF treatment for 1 h, nuclear extracts were prepared, and chromatin immunoprecipitation (ChIP) (upper panel) and DNA affinity precipitation (lower panel) assays were performed as described in “Materials and methods”. Binding of p65 and c-Jun to the AP1 site of PTX3 promoter region (AP1 (wt)) and mutant probe (AP1 (mut)) was analyzed by Western blotting. HDAC1, loading control. (C) KB cells were transfected with 20 nM c-Jun siRNA oligonucleotides by lipofection. After 50 ng/ml EGF treatment for 6 h, expressions of PTX3, c-Jun, and α-tubulin were analyzed by Western blotting (WB). (D) KB cells were transfected with 0.5 μg PTX3 promoter construct, 1 μg DN-IκB expression vector, and 1 μg pRSV-c-Jun by lipofection. Luciferase activities and protein concentrations were then determined and normalized. Values are represented as the mean ± S.E. of three determinations. (E) Parental (wt) and shRelA cells were treated with 50 ng/ml EGF for 6 h. Expressions of p65, c-Jun and phosphorylation of c-Jun on Ser73 were analyzed by WB. shLacZ, negative control. HDAC1, loading control.

Our results showed that the PI3K/Akt and NF-κB pathways are involved in EGF-induced expression of PTX3. To further clarify the response element of EGF-induced promoter activity and verify the binding of NF-κB to the promoter that is essential for regulating PTX3 mRNA induction, the promoter region of PTX3 bearing the mutated NF-κB-binding site (NF-κB mut) was subcloned into the luciferase-based reporter system. In addition, the predicted Sp1- and AP1-binding sites were also mutated and subcloned. The binding of Sp1, NF-κB, and AP1 to their respective sites and their binding specificities were confirmed by a DNA affinity precipitation assay. Transcription factors lost their binding ability to the PTX3 promoter in which Sp1, NF-κB, and AP1 sequences were mutated (Supplementary Fig. 4). Next, we clarified the response element of EGF-induced PTX3 promoter activity. As shown in Supplementary Fig. 5, although the predicted Sp1 and NF-κB sites were deleted, the multiples of induction induced by EGF were almost unchanged in the promoter constructs PTX3-473 and PTX3-44 compared to PTX3-1200. The requirement of Sp1-, AP1-, and NF-κB-binding sites for EGF-induced promoter activity was also verified using mutant PTX3 promoter constructs. Compare to the wild-type (WT) PTX3-1200, the EGF also triggered the activation of the PTX3-1200-Sp1 M and PTX3-1200-NF-κB M constructs (Fig. 3A). However, the basal activity of the promoter was reduced in the PTX3-1200-Sp1 M construct, which resulted in a decreased relative intensity of EGF-mediated promoter activity (Fig. 3A). In addition, IL-1β-induced promoter activity was inhibited in the PTX3-1200-NF-κB M construct (Supplementary Fig. 6), suggesting that the NF-κB-binding site is essential for cytokine-induced PTX3 expression. These results indicated that the Sp1 and NF-κB sites in the promoter region of −1200 to −44 bp are not essential for EGF-induced PTX3 promoter activity. To investigate whether binding sites within the −44 to +60-bp promoter region are required for EGF-induced PTX3 promoter activity, PTX3-1200-AP1 M, PTX3-1200-PEA3 M, and PTX3-1200-AP1/PEA3 M constructs were used. As shown in Fig. 3A, the multiples of induction of EGF-induced promoter activity were significantly reduced in the PTX3-1200-AP1 M and PTX3-1200-AP1/PEA3 M constructs but only slightly reduced in the PTX3-1200-PEA3 M construct. These results indicated that the AP1-binding site located in the −33 to −27-bp region is essential for the EGF response.

Because the AP1-binding site, but not the NF-κB-binding site, is essential for EGF-induced PTX3 promoter activity, we further investigated whether EGF-stimulated binding of NF-κB to the PTX3 promoter was through the AP1-binding site. Increased binding of c-Jun and NF-κB to the PTX3 promoter in cells treated with EGF was observed *in vivo* using a chromatin precipitation assay (Fig. 3B). As shown in Fig. 3B, EGF enhanced the binding of c-Jun and NF-κB to DNA. However, the binding of c-Jun but not NF-κB was significantly reduced with the AP1 mutant probe. In addition, the EGF-induced binding of NF-κB to DNA did not decrease in c-Jun-knockdown cells (Fig. 3B). However, knock down of both NF-κB (Fig. 2B) and c-Jun (Fig. 3C) significantly inhibited EGF-induced PTX3 expression. In order to further confirm the cooperation between c-Jun and NF-κB in EGF-induced PTX3 expression, the effect of DN-IκB overexpression on c-Jun-induced PTX3 promoter activity was analyzed. As shown in Fig. 3D, DN-IκB dramatically inhibited c-Jun-induced PTX3 promoter activity. In addition, NF-κB knockdown and inhibition of NF-κB activation dramatically inhibited the EGF-induced expression and activation of c-Jun (Fig. 3E). These results suggested that, although the binding of NF-κB to the promoter is not involved in the regulation of PTX3 promoter activity, the cooperation between c-Jun and NF-κB is essential for EGF-induced PTX3 expression.

### Expression of PTX3 contributes to EGF-induced tumor cell migration and invasion

**Figure 4:**
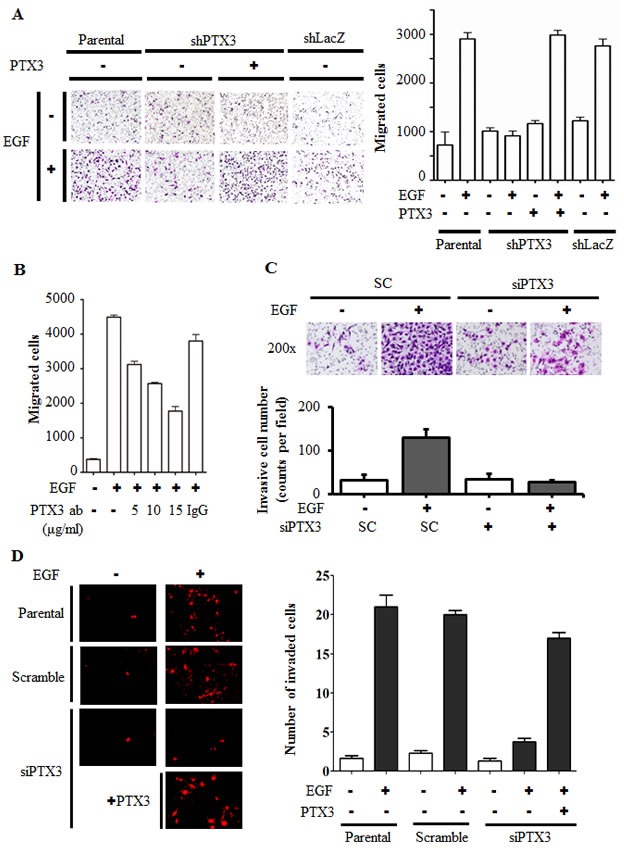
EGF-induced PTX3 enhances tumor migration and invasion (A) The migratory properties of KB cells were analyzed by a transwell assay using Boyden chambers. Parental and shPTX3 KB cells were incubated with or without 50 ng/ml EGF and 250 ng/ml PTX3 recombinant protein in the upper chamber of filter inserts. After incubation for 15 h, non-migratory cells were removed, and migratory cells were fixed and stained by a crystal violet procedure. Original magnification, ×200 (left panel). Migrating cells were counted (right panel). Values represent the mean ± S.E. of three determinations. shLacZ, negative control. (B) HONE1 cells were treated with various concentrations of anti-PTX3 antibodies (ab), 15 μg/ml immunoglobulin G (IgG) and 50 ng/ml EGF for 15 h. Migratory cells were fixed and stained by a crystal violet procedure. Migrating cells were counted. Values represent the mean ± S.E. of three determinations. (C) The invasive properties of HONE1 cells were examined using invasion assay as described in “Materials and methods”. Cells were transfected with 30 nM PTX3 siRNA oligonucleotides and scrambled oligonucleotides (SC) by lipofection. After 50 ng/ml EGF treatment for 48 h, the non-invasive cells were removed and invasive cells were fixed and stained by crystal violet procedure. The invasive images were examined using a microscope (upper panel). The number of invasion cells was counted as shown in lower panel. Values represent the mean ± S.E. of three determinations. (D) Endothelial cells were grown to form a monolayer on the bottom of a thick layer of extracellular matrix proteins to mimic intravasation in transendothelial invasion assays. HONE1 cells were transfected with 30 nM PTX3 siRNA oligonucleotides by lipofection. After 50 ng/ml EGF treatment for 6 h and stained with DiI, cells were loaded in the upper chamber of filter inserts. After incubation for 48 h, non-invasive cells were removed. The invasive images were examined using a microscope (left panel). Original magnification, ×200. Invasive cells were counted (right panel). Values represent the mean ± S.E. of three determinations.

In mice, the PTX3 null mutation is associated with a severe defect in female fertility caused by an abnormal location of granulosa cells. An abnormal cumulus oophorus is due to defective organization and stability of the ECM [22]. Thus, we raised the hypothesis that PTX3 may also participate in tumor migration. To investigate the biological significance of EGF-induced PTX3 expression by tumor cells, stable cell lines with PTX3-knockdown via shRNA knockdown of PTX3 (shPTX3) was used. As shown in Supplementary Fig. 7, EGF-induced PTX3 mRNA expression and protein secretion into culture media were dramatically inhibited in shPTX3 cells. These shPTX3 cells were then used to study the effects of EGF on cell migration. As shown in Fig. 4A, EGF induced cancer cell migration in parental but not PTX3-knockdown cells. However, recombinant PTX3 abolished this inhibition in EGF-treated shPTX3 cells. Again, depletion of PTX3 using PTX3 siRNA oligonucleotides significantly blocked EGF-induced cell migration (Supplementary Fig. 8). The involvement of PTX3 in regulating EGF-induced migration was further examined using anti-PTX3 antibodies to neutralize PTX3 in culture media. Anti-PTX3 antibodies blocked EGF-induced cell migration in a dose-dependent manner (Fig. 4B). In addition, we studied whether PTX3 contributed to EGF-induced tumor invasion. Indeed, depletion of PTX3 inhibited EGF-induced HNSCC cell invasion (Fig. 4C). We further studied the effect of PTX3 on the endothelial transmigration of tumor cells in a three-dimensional *in vitro* model. Briefly, endothelial cells were grown until they formed a monolayer on the bottom of a thick layer of ECM proteins to mimic intravasation in transendothelial assays. As shown in Fig. 4D and Supplementary Fig. 9, EGF-induced transendothelial invasion was significantly reduced in PTX3 knockdown cells. Addition of PTX3 recovered the EGF-induced transeddothelial invasion in PTX3 knockdown cells (Fig. 4D).

**Figure 5:**
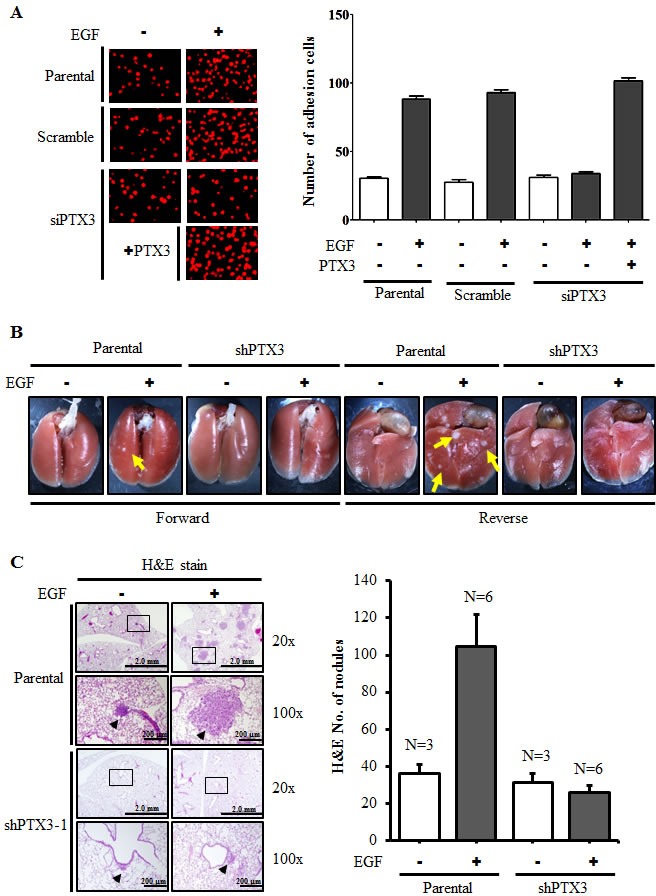
PTX3 mediates EGF priming for tumor dissemination to the lungs (A) FaDu cells were transfected with 30 nM PTX3 siRNA oligonucleotides by lipofection. After 50 ng/ml EGF and 250 ng/ml PTX3 treatment for 3 h, cells were then labeled with DiI and cultured with endothelial cells for 3 h. The attachment of cells was examined using a microscope (left panel). The number of attached cells was counted using three randomly chosen fields under the microscope from three independent experiments (right panel). Values represent the mean ± S.E. of three determinations. (B) A lung-colonization analysis was performed by injecting 2 × 10^5^ FaDu cells into a lateral tail vein of mice. Prior to the injection, cells were treated as indicated with 50 ng/ml EGF for 3 h. Nodules were examined and photographed at 2 month. Arrows point to metastatic nodules. (C) The images of tumors (left panel) and numbers of nodules (right panel) were examined using H&E staining and counted under a microscope, respectively. Error bars indicate SEM. N, number of SCID mice.

During tumor metastasis, the infiltration of tumor cells to distant destinations relies on their attachment to blood vessels. To test the possibility that the metastatic process enhanced by EGF-induced PTX3 also occurs by regulating the interaction between tumor and endothelial cells, we examined whether EGF induces the binding of HNSCC cells to HMEC-1 endothelial cells. As shown in Fig. 5A, EGF promoted the binding of HONE1 cells to HMEC-1 cells, and this binding was dramatically blocked in PTX3-knockdown condition. The inhibition of this tumor-endothelial cell interaction was rescued when PTX3-knockdown cells were treated with EGF and PTX3 (Fig. 5A). These results indicated that the autocrine production of EGF-induced PTX3 stimulated the binding of tumor cells to endothelial cells, which may result in an enhancement of the ability of tumor cells to penetrate blood vessels. In addition, to study whether EGF-induced PTX3 regulated the distal dissemination of tumor cells, EGF-treated parental and shPTX3 cells were injected into the tail vein of mice. As shown in Fig. 5B and C, an increase in metastatic nodules in lung tissues was found in EGF-treated parental cells. Consistent with what was observed in the transendothelial invasion assay, depletion of PTX3 inhibited metastatic seeding of EGF-primed tumor cells in the lungs (Fig. 5B and C). These results indicated that EGF primed tumor cells for metastatic seeding of the lungs by induction of PTX3 expression.

### EGF-induced PTX3 activates the fibronectin signaling to improve invasion

**Figure 6:**
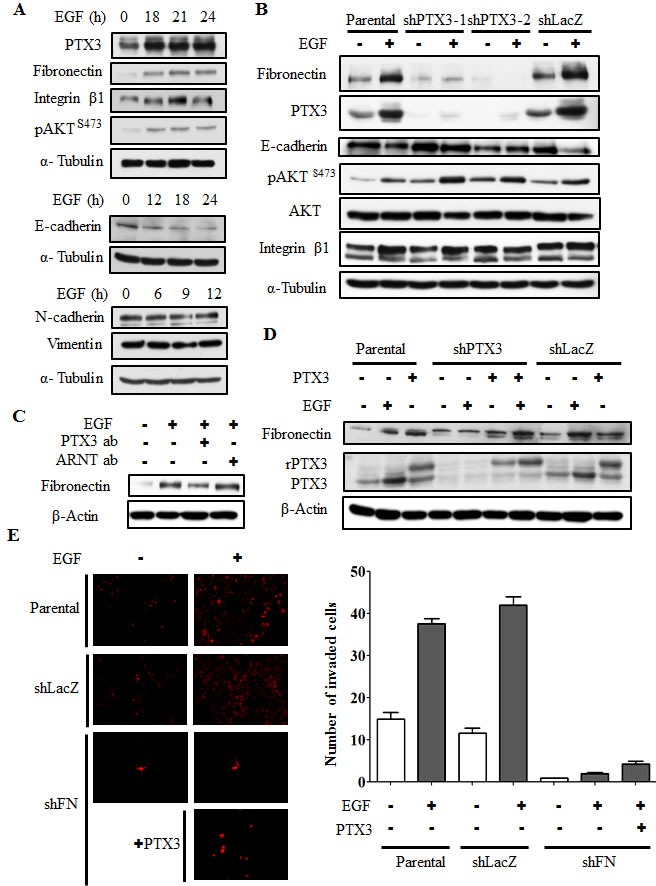
EGF-induced PTX3 regulates expressions of fibronectin and E-cadherin (A) HONE1 cells were treated with 50 ng/ml EGF for a period of time as indicated. Lysates of cells were prepared and subjected to SDS-PAGE and analyzed by Western blotting with antibodies against PTX3, fibronectin, integrin β1, phosphorylation of Akt at serine 473, E-cadherin, N-cadherin, vimentin, and α-tubulin. (B) Parental and shPTX3 HONE1 cells were treated with 50 ng/ml EGF. Lysates of cells were prepared and subjected to SDS-PAGE and analyzed by Western blotting with antibodies against PTX3, fibronectin, phosphorylation of Akt at serine 473, E-cadherin, integrin β1, and α-tubulin. shLacZ, negative control. (C) HONE1 cells were treated with 50 ng/ml EGF and 15 μg/ml anti-PTX3 antibodies or 15 μg/ml aryl hydrocarbon receptor nuclear translocator (ARNT) antibodies as negative control. Lysates of cells were prepared and subjected to SDS-PAGE and analyzed by Western blotting with antibodies against fibronectin and β-actin. (D) Parental and shPTX3 HONE1 cells were treated with 50 ng/ml EGF and 250 ng/ml PTX3 protein. Lysates of cells were prepared and subjected to SDS-PAGE and analyzed by Western blotting with antibodies against PTX3, fibronectin and β-actin. shLacZ, negative control. (E) Transendothelial invasion assay was performed as described in “Material and methods”. Parental and fibronectin knockdown (shFN) HONE1 cells were treated with 250 ng/ml PTX3 and 50 ng/ml EGF for 6 h and stained with DiI, and then were loaded in the upper chamber of filter inserts. After incubation for 48 h, the non-invasive cells were removed. The invasive images were examined using a microscope (left panel). Original magnification, ×200. The number of invasion cells was counted using three randomly chosen fields under the microscope from three independent experiments (right panel). Error bars indicate SEM. shLacZ, negative control.

**Figure 7:**
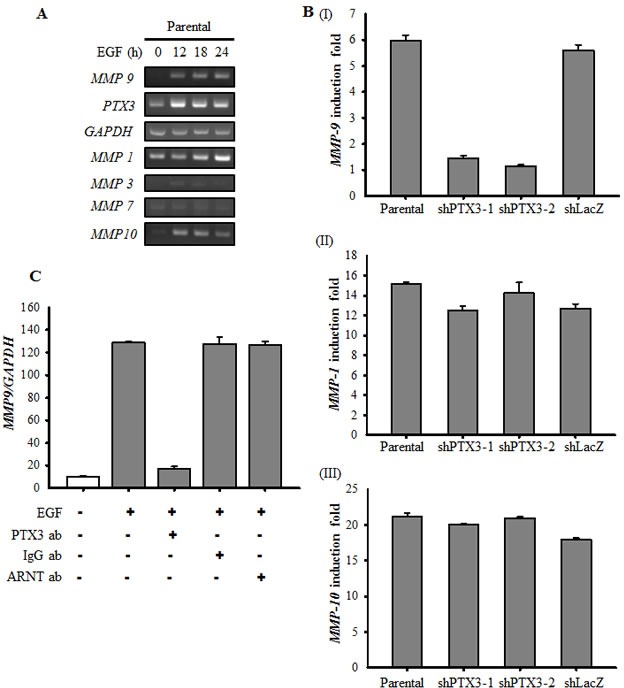
EGF-induced PTX3 regulates the expression of MMP9 (A) HONE1 cells were treated with 50 ng/ml EGF for a period of time as indicated. Expressions of *PTX3, MMP1, MMP3, MMP7, MMP9, MMP10* and *GAPDH* mRNA were analyzed by RT-PCR and examined in 2% agarose gel. (B) Parental and shPTX3 HONE1 cells were treated with 50 ng/ml EGF for 6 h. The expression of *MMP9* (I), *MMP1* (II) and *MMP10* (III) mRNA was also analyzed by Real-time quantitative PCR. Relative levels of *MMPs* were normalized by *GADPH*. Error bars indicate SEM of three independent experiments. shLacZ, negative control. (C) NONE1 cells were treated with 15 μg/ml anti-PTX3 antibodies, 15 μg/ml immunoglobulin G (IgG), 15 μg/ml anti-aryl hydrocarbon receptor nuclear translocator (ARNT) antibodies and 50 ng/ml EGF for 9 h. The expression of *MMP9* mRNA was also analyzed by Real-time quantitative PCR. Relative levels of *MMP9* were normalized by *GADPH*. Error bars indicate SEM of three independent experiments.

Based on the observation that PTX3 expression is essential for EGF-enhanced cancer metastasis, we next clarified the mechanisms involved in PTX3-regulated cell metastasis. The expressions of fibronectin and integrin β1, and the phosphorylation of Akt increased in EGF-treated cells (Fig. 6A). In addition, EGF inhibited the expression of E-cadherin, but had no effect on vimentin or N-cadherin expressions (Fig. 6A). Although no changes in EGF-induced integrin β1 or phospho-Akt were observed in shPTX3 cells, EGF-regulated expressions of fibronectin and E-cadherin were significantly inhibited in PTX3-knockdown cells (Fig. 6B). EGF-induced expression of fibronectin was also inhibited when PTX3 was neutralized by anti-PTX3 antibodies (Fig. 6C). In addition, repression of fibronectin expression in shPTX3 cells was abolished when cells were treated with EGF and PTX3 (Fig. 6D). To further confirm the function of fibronectin for tumor invasion in EGF-treated cells, we study the transendothelial invasion in EGF- and PTX3-treated fibronectin knockdown cells. As shown in Fig. 6E, even in cotreatment with PTX3, EGF-induced invasion was blocked in loss-of-fibronectin. These results suggested that the induction of PTX3/fibronectin axis was essential for EGF-induced tumor invasion. The activation of the Rac1/cdc42 was a key regulator downstream of fibronectin to mediate cell migration. We examined whether EGF-activated Rac1/cdc42 is regulated by fibronectin expression. We found that EGF-induced phosphorylation of Rac1/cdc42 was significantly inhibited in the depletion of fibronectin (Supplementary Fig. 10A). Again, siRNAs targeted fibronectin also inhibited EGF-induced cell invasion (Supplementary Fig. 10B). To further investigate whether EGF-regulated MMP expressions in HNSCC are due to the induction of PTX3, expressions of MMPs were examined in PTX3-depleted cells. As shown in Fig. 7A and 7B, the expression of EGF-induced MMP9 was repressed in PTX3-knockdown cells. However, no changes in EGF-induced MMP1 or MMP10 were observed in shPTX3 cells. EGF-induced expression of MMP9 was also inhibited when PTX3 was neutralized by anti-PTX3 antibodies (Fig. 7C). Taken together, these results demonstrated that the EGF/PTX3/fibronectin axis is an important pathway in regulating HNSCC migration and invasion.

## DISCUSSION

Activation of EGFR pathways is critical in driving tumor invasion. In particular, the EGFR is expressed at a high level in HNSCC, therefore identifying downstream molecules might provide diagnostic markers for the invasiveness of HNSCC. Herein, we found elevated gene expression of PTX3 in malignant and metastatic HNSCC tissues according to the cancer microarray database (Oncomine 4.0). In addition, EGF-induced PTX3 expression was through the activation of PI3K/Akt and NF-κB pathways in HNSCC. The autocrine production of EGF-induced PTX3 altered the expression of metastatic molecules, such as increasing fibronectin and MMP-9, resulting in enhanced tumor metastasis. These observations strongly indicate that PTX3 plays an important role in the invasion of HNSCC.

In the tail vein injection mice model, tumor nodules were significantly produced in lung tissues after an injection of parental but not shPTX3 cells. These results indicate that the EGF primes tumor cells for metastatic seeding of the lungs by inducing PTX3 expression. Consistent with these results, growth factors such as TGF-β induce ANGPTL4 expression to prime the attachment of tumor cells to microvessels, resulting in metastatic lung colonization [23]. Those studies suggested that the extravasation process primed by growth factor-induced proteins such as PTX3 and ANGPTL4 is a limiting step for tumor distal dissemination. In addition, EGF-induced PTX3 also enhanced tumor cell-endothelial cell interactions. Thus, we speculate that EGF-induced PTX3 promoted tumor metastasis by modulating adhesion between tumor and endothelial cells. These results also indicate that disruption of adhesion signaling axes between endothelial cells and tumor cells may serve to prevent dissemination of metastatic HNSCC. Therefore, it would be interesting to further pursue the potential functions of PTX3 in mediating extravasation.

The PTX3 promoter region contains a number of potential transcription factors binding sites, including NF-IL-6, NF-κB, AP1, PEA3, and Sp1 and no obvious TATA or CAAT consensus sequences were found [24]. In regulating PTX3 gene expression, PTX3 promoter activity is strongly induced by IL-1 and TNF-α through the NF-κB-binding site [24]. Although NF-κB activation is essential for EGF-induced PTX3 expression, the promoter activity was regulated by the binding of c-Jun, but not NF-κB to the AP1-binding site. These results indicate that cooperation of c-Jun and NF-κB was specifically responsive to individual cytokine- and EGF-regulated PTX3 expression. In general, EGF-regulated gene expression is dependent on activation of downstream effectors, such as the PI3K/Akt, MEK/ERK, and JAK/STAT signaling pathways [25]. According to our results, Akt/NF-κB was the main pathway for EGF-induced PTX3 gene expression. The EGF-mediated JNK and ERK expression were not correlated with induction of PTX3 expression. These results were consistent with the concept that activation of the NF-κB pathway is critical for the expression of cytokines [26]. In addition to NF-κB being essential for PTX3 gene expression, EGF also regulates IL-1β, IL-6, and IL-8 expressions through activation of STAT-3 and NF-κB [21, 27, 28]. The importance of NF-κB activation is evidenced by the blocking of EMT elicited by nondestructible IκB, an NF-κB inhibitor [29]. In addition, we found that EGF-induced PTX3 expression was inhibited by DN-IκB. These results suggest that the increase in PTX3 by EGF-induced activation of the NF-κB pathway contributes to NF-κB-promoted tumor metastasis.

The EGFR inhibitor, cetuximab, plus radiation therapy improves the overall survival of patients without observable metastasis in current treatment of HNSCC [30]. Unfortunately, owing to drug resistance, few patients with metastasis are responsive to cetuximab, the only EGFR-targeted therapy approved for HNSCC [30, 31]. The downstream target of the EGFR that enhances HNSCC metastasis remains unclear. In addressing this challenge, our work links the autocrine production of PTX3 by activating EGFR signaling to regulate fibronectin and MMP-9 expressions, resulting in promotion of HNSCC metastasis. Identifying the molecules involved in the regulation of EGF-regulated HNSCC metastasis should be able to improve the chemotherapeutics when applying EGFR inhibitors. Therefore, the combination of EGFR inhibitors and antibodies to neutralize PTX3 could possibly prevent HNSCC metastasis. In addition, recent reports suggested that EGF-mediated store-operated calcium signaling is involved to promote cancer cell invasion and metastasis in various cancers [32-35]. Both NSAID and microRNA-185 have been reported to prevent calcium-dependent cancer cell metastasis [35, 36]. Thus, it would be an important issue to determine the association between dysregulated calcium signals, PTX3 activation and cancer prognosis.

In conclusion, we demonstrated the clinical and biological functions of PTX3 in HNSCC. Through measurement of PTX3 expression levels might provide prognostic information about HNSCC metastasis, which leads PTX3 a novel therapeutic target for EGFR-overexpressing HNSCC.

## MATERIALS AND METHODS

### Cell culture

Cell lines of head and neck cancer (*SCC25, KB and FaDu*) were purchased from American Type Culture Collection (ATCC, Manassas, VA, USA). Head and neck cancer cells (HONE1, TU183 and SAS) and human microvascular endothelial cell line (HMEC-1) were kindly provided by Dr. Kwang-Yu Chang (National Health Research Institutes, Taiwan) [37] and Dr. Trai-Ming Yeh (Department of Medical Laboratory Science and Biotechnology, Medical College, National Cheng Kung University), respectively. Cell culture condition was performed as previously described [37, 38].

### Western blotting

An analytical 12% SDS-PAGE was performed, and 30 μg of protein of each were analyzed, unless stated otherwise. Western blotting was performed as previously described [39]. Antibodies against human phospho-Akt^S473^, phospho-IκBα^S32/36^ and phospho-ERK1/2^T202/Y204^ (Cell Signaling Technology, Danvers, MA); PTX3, IκBα, NF-κB p65, Fibronectin, E-cadherin, Vimentin and ERK1/2 (Santa Cruz Biotechnology, Inc., Santa Cruz, CA); c-Jun (BD Biosciences, Franklin Lakes, NJ); N-cadherin and Integrin β1 (Abcam, Cambridge, MA); Sp1 (Millipore, Billerica, MA), and α-Tubulin (Sigma, St. Louis, MO) were used as the primary antibodies.

### Reverse transcription-PCR and real-time quantitative RT-PCR

Total RNA was isolated using the TRIzol RNA extraction kit (Invitrogen), and 1 μg of RNA was subjected to reverse transcription-PCR with ImProm-II™ (Promega). The *PTX3* specific primers (sense, 5′-CATCCAGTGAGACCAATGAG-3′; antisense, 5′-GTAGCCGCCAGTTCACCATT-3′), *MMP1* specific primers (sense, 5′-ATGCACAGCTTTCCTCCACT-3′; antisense, 5′-TTCCCAGTCACTTTCAGCCC-3′), *MMP3* specific primers (sense, 5′-GCAAGACAGCAAGGCATAGAG-3′; antisense, 5′-CCGTCACCTCCAATCCAAGG-3′), *MMP7* specific primers (sense, 5′-GCTACAGTGGGAACAGGCTC-3′; antisense, 5′-TGGCCCATCAAATGGGTAGG-3′), *MMP9* specific primers (sense, 5′-GACAAGAGCCAGGAAGAAACC-3′; antisense, 5′-CTTTAGCACTCCTTGGCAAAAC-3′), *MMP10* specific primers (sense, 5′-GTTTGACCCCAATGCCAGGA-3′; antisense, 5′-TCTGCAAGGCTCATCTTCTTC-3′), *JNK2* specific primers (sense, 5′-GGCGCCCGAAGTCATCCTGG-3′; antisense, 5′-ACTGAGAAGAGTGGCGTTGCT-3′), *MEK1* specific primers (sense, 5′-TCGCAGAGCGGCTAGGAGCA-3′; antisense, 5′-AACGCGCTTCCAACTCCGGG-3′), *RelA* specific primers (sense, 5′-AGCAGCGTGGGGACTACGAC-3′; antisense, 5′-AGGCTGGGGTCTGCGTAGGG-3′), *IκB* specific primers (sense, 5′-ATGGTCAAGGAGCTGCAGGAGATC-3′; antisense, 5′-TCATAACGTCAGACGCTGGCCTC-3′) and *glyceraldehyde-3-phosphate dehydrogenase* specific primers (sense, 5′-CCATCACCATCTTCCAGGAG-3′; antisense, 5′-CCTGCTTCACCACCTTCTTG-3′) were used. The PCR products were separated by 2% agarose gel electrophoresis and visualized with ethidium bromide staining. For the quantitative real-time RT-PCR, cDNA synthesis was performed using the TITANIUM One-Step TR-PCR kit (Clontech) containing SYBR Green I. Real-time fluorescence monitoring and the melting curve analysis were performed with LightCycler according to the manufacturer`s recommendations. The relative transcript amount of the target gene, calculated using standard curves of serial RNA dilutions, was normalized to that of glyceraldehydes-3-phosphate dehydrogenase (GAPDH) of the same RNA.

### Knockdown experiments

RNA interference vectors used in this study were obtained from the National RNAi Core Facility in the Institute of Molecular Biology, Academia Sinica (Taipei, Taiwan). The lentivirus of hairpins targeting RelA (shRelA), PTX3 (shPTX3), MEK1 (shMEK1), JNK2 (shJNK2) and fibronectin 1 (shFN) were obtained from RNAi Core of National Cheng Kung University Hospital in the pLKO.1 lentiviral backbone. Cells were selected by treating with 2 μg/ml puromycin.

### Enzyme-linked immunosorbent assay

Quantitation of the secretion of PTX3 in the *culture medium* was achieved by an Enzyme-linked immunosorbent assay (ELISA) according to the manufacturer's (R&D Systems). Briefly, after EGF treatment, 100 μl of culture medium was collected and incubated with pre-coated capture antibody at room temperature in a 96-well microplate. After washing step, 100 μl of detection antibody was added and incubated for another 2 h at room temperature. Add 100 μl of the working dilution of Streptavidin-HRP to each well and incubated for 20 min under protecting from light. After washing step, 100 μl of substrate solution was added and incubated for 20 min under protecting from light. After adding 50 μl of stop solution to each well, gently tapped the plate to ensure thorough mixing and used a microplate reader to quantify the optical density at 450 nm immediately.

### Plasmid construction

The DNA fragment bearing the promoter region of PTX3 (1200 bp) was kindly provided by Dr. Wang JM [14]. The mutants were constructed by the site-directed mutagenesis method. Synthetic primers were shown in the following: pPTX3-Sp1M mutant primer 5′-CCCTCGCTCTCCTATCTAGCTCTTTCTCCC ATCAAATTCAGGGG-3′; pPTX3-NF-κBM mutant primer 5′-CCTCCCCCACCAAATTCAATTCTAGACCC GTTACCGCAGTGCCAC-3′; pPTX3-PEA3M mutant primer 5′-CCATTCAGGCTCCCATCAGCATTTATTAAGG-3′ and pPTX3-AP1M mutant primer 5′-GCAGTGCCACCAGCACACACTTTTCAT CTCCATTCAGGCTTTCC-3′. Mutated positions in the sequence of the primers were underlined. The vector sequence was confirmed by DNA sequencing. Dominant negative IκB mutant was generated by N-terminal deletion of residues 1-45 using a standard PCR approach [21]. pTK minimal promoter with 5 repeated NF-κB biding sites was generated by PCR and subcloned into pGL3 basic vector (Promega).

### DNA transfection and luciferase assay

Transient transfection of cells with plasmids was performed with Lipofectamine 2000 (Invitrogen) according to the manufacturer's instructions but with slight modification. Cells were replated 36 h before transfection at a density of 3 × 10^5^ cells in 2 ml of fresh culture medium in a 3.5-cm plastic dish. For use in transfection, 2 μl of Lipofectamine 2000 was incubated with 0.5 μg of luciferase reporter plasmid in 1 ml of Opti-MEM medium for 30 min at room temperature. Cells were transfected by changing the medium with 1 ml of Opti-MEM medium containing the plasmids and Lipofectamine 2000 and then incubated at 37 °C in a humidified atmosphere of 5% CO_2_ for 24 h. The luciferase activity in cell lysate was determined as described previously [39].

### Chromatin immunoprecipitation (ChIP) assay

Chromatin immunoprecipitation assay was performed as previous reported [40] with minor modifications. Briefly, cells were treated with 1% formaldehyde for 15 minutes. The crosslinked chromatin was sonicated to 400-500 bp fragments. Lysates were precleaned with protein A beads and incubated overnight at 4°C with antibodies specific to c-Jun, p65 or control rabbit IgG. Immune complexes were precipitated with protein A beads pre-absorbed with sonicated ssDNA and BSA. After reversal of cross-linking, levels of precipitated PTX3 promoter DNA were determined by PCR. Oligonucleotides spanning the AP1 binding site of PTX3 were as follows: sense, 5′-GGGAAGTGTT CTCATCTATG-3′; antisense, 5′-GGCCACTTCCAACCTTreTAAA-3′. The PCR products were separated by 1% agarose-gel electrophoresis and visualized with ethidium bromide staining.

### DNA affinity precipitation assay

Quantitation of the change of c-Jun and NF-κB binding to the *PTX3* promoter element was achieved by a DNA affinity precipitation assay according to the method previously described [21]. In brief, 5′-biotinylated oligonucleotides and corresponding to the sense −14 to −42 bp and antisense strands of the *PTX3* promoter element were annealed. The DNA affinity precipitation assay was performed by incubating 2 μg of biotinylated DNA probe with 200 μg of nuclear extract and 20 μl of streptavidin-agarose beads in phosphate-buffered saline at room temperature for 1 h with rotation. Beads were collected and washed three times with cold phosphate-buffered saline. The binding proteins were eluted by loading buffer and separated by SDS-PAGE, followed by Western blot analysis probed with specific antibodies.

### Migration and invasion assays

Both assays were performed using Millicell™ hanging cell culture inserts (polyethylene terephthalate (PET) membranes with 8 μm pores) (Millipore). For the transwell migration assay, 2 × 10^5^ cells were plated in serum-free medium containing 50 ng/ml EGF (Invitrogen) and placed in the upper chamber for 15 h, while the lower chamber was filled with serum-free medium with or without 50 ng/ml EGF. The cells in the upper chamber were removed and the migrated cells at the bottom of the PET membrane were fixed with 4% paraformaldehyde and stained with 0.1% crystal violet. For the invasion assay, 2 × 10^5^ cells were plated in serum-free medium containing 50 ng/ml EGF and placed in the upper chamber on a 10% Matrigel-coated membrane, while the lower chamber was filled with serum-free medium. After incubation for 48 h, the cells in the upper chamber were removed and the invaded cells at the bottom of the PET membrane were fixed with 4% paraformaldehyde and stained with 0.1% crystal violet. In both assays, the number of invading cells was determined in three randomly chosen fields under the microscope for three independent experiments.

### Transendothelial invasion assay

The invasion assay was performed using Millicell™ hanging cell culture inserts (polyethylene terephthalate (PET) membranes with 8 μm pores) (Millipore). HMEC-1 cells (1 × 10^5^ cells per well) were plated on the upper chamber and allowed to grow to confluence, and then 10% Matrigel was loaded into the chamber. Tumor cells were treated with 50 ng/ml EGF in serum-free medium and then stained with 1,1′-dioctadecyl-3,3,3′,3′-tetramethyl-indocarbocyanine perchlorate (*DiI)* (Invitrogen) for 30 min. DiI-stained tumor cells (2 × 10^5^) were then loaded into the chamber, which was filled with serum-free medium, and incubated for 2 days. Cells on the apical side of each insert were scraped off. Invasion to the basolateral side of the membrane was visualized using an immunofluorescent microscope. The number of invading cells was determined in three randomly chosen fields under the microscope for three independent experiments.

### Immunofluorescence

Immunofluorescence was performed as previously described [39]. Cells grown on chamber slides were treated with 50 ng/ml EGF for a period of time. Cells were fixed with 4% paraformaldehyde (Sigma) in phosphate-buffered saline at 4 °C for 10 min. The cells were then rinsed with phosphate-buffered saline three times and permeabilized with 1% Triton X-100 for 7 min. Next, the cells were pretreated with 1% bovine serum albumin in phosphate-buffered saline at 25 °C for 60 min and incubated with indicated antibody at a dilution of 1:100 for 1 h and treated with fluorescein isothiocyanate-conjugated donkey anti-rabbit IgG polyclonal antibodies (Jackson ImmunoResearch Laboratories, West Grove, PA) at a dilution of 1:250 for 1 h. Finally, the cells were washed with phosphate-buffered saline, mounted in 90% glycerol containing 4,6-diamidino-2-phenylindole (DAPI) (Invitrogen), and examined by using a microscope (model DMI 4000 B; Leica).

### Cell adhesion assay

Briefly, tumor cells were treated with 50 ng/ml of EGF or 250 ng/ml of PTX3 for 3 h then labeled for 30 min at 37 °C with DiI (Invitrogen) and washed twice with phosphate-buffered saline. The medium was removed from the wells, and tumor cells (1.5 × 10^5^ cells /ml) were added to a monolayer of HMEC-1 cells. After incubation for 30 min at 37 °C, the wells were gently washed twice with phosphate-buffered saline to remove non-adherent cells. The cells were photographed and numbers were quantified under a fluorescence microscope.

### Tumor metastasis assay in animal model

Tumor metastasis was determined by tail vein intravenous injection of cancer cells into 4- to 6-week-old male severe combined immunodeficiency (SCID) mice. Briefly, each animal was injected with 1 × 10^6^ cells mixed with phosphate-buffered saline, and all mice were sacrificed until 2 month after injection. All mice were obtained from the National Cheng Kung University Laboratory Animal Center (Tainan, Taiwan) and the National Laboratory Animal Center (Tainan, Taiwan). All animal experiments in this study were approved by the Laboratory Animal Committee of National Cheng Kung University. H&E stained were performed by the Human Biobank, Research Center of Clinical Medicine, National Cheng Kung University Hospital.

### Statistical analysis

Data were expressed as mean + SEM. Statistical analysis was performed using GraphPad Prism 5 statistical software (La Jolla, CA, USA) for Microsoft Windows.

Supplementary information accompanies the paper on the Oncotarget website.

## SUPPLEMENTARY MATERIAL


