# Supplementary material for: PTX3 gene activation in EGF-induced head and neck cancer cell metastasis
Source: Oncotarget. 2015 Mar 8;6(10):7741–57. doi: 10.18632/oncotarget.3482 (PMC4480713; doi:10.18632/oncotarget.3482)
Supplement: Supplementary file 1 [file oncotarget-06-7741-s001.pdf]

PTX3 gene activation in EGF-induced head and neck cancer cell metastasis

Supplementary Material

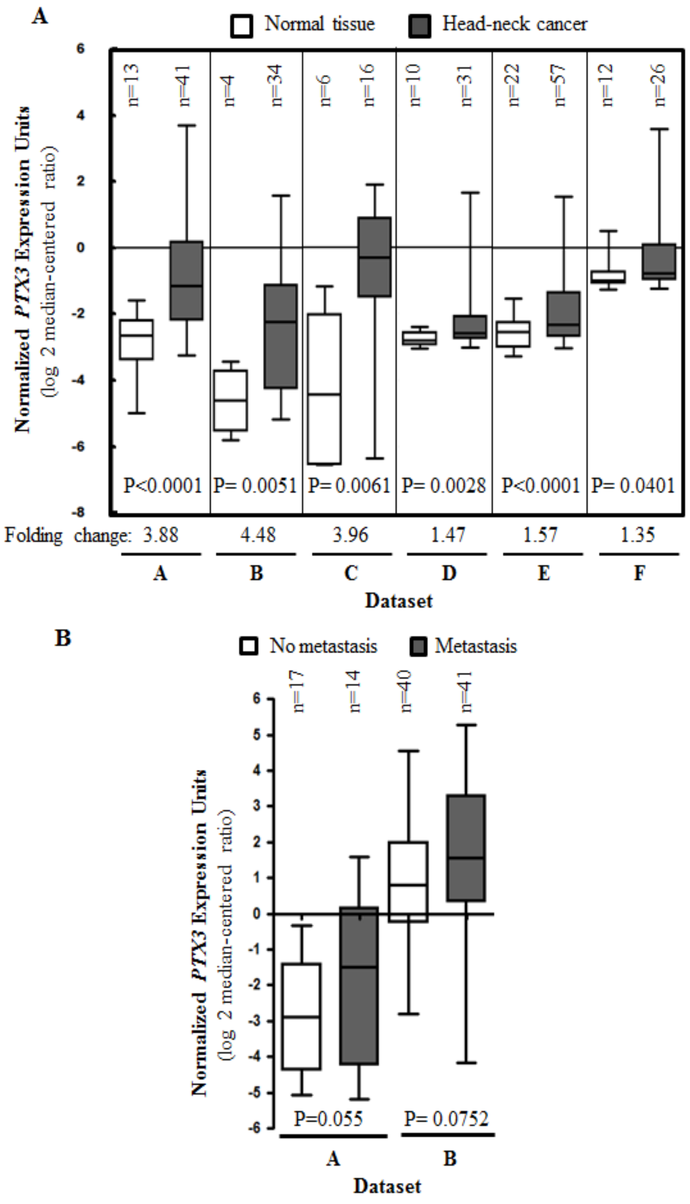

Supplementary Figure 1: PTX3 is upregulated in HNSCC. (A) Data mining was performed on the cancer microarray database Oncomine 4.0 (Oncomine DB at www.oncomine.org). Oncomine boxed plot of *PTX3* expression levels between human normal prostate gland and head and neck cancer in multiple datasets from ref. 1 (A), ref. 2 (B), ref. 3 (C), ref. 4 (D), ref. 5 (E), and ref. 6

(F). 1. Ginos MA, et al. Cancer Res 2004;64:55-63. 2. Cromer A, et al. Oncogene 2004;23:2484-98. 3. Frierson HF Jr, et al. Am J Pathol 2002;161:1315-23. 4. Sengupta S, et al. Cancer Res 2006;66:7999-8006. 5. Peng CH, et al. PLoS One 2011;6:e23452. 6. Ye H, et al. BMC Genomics 2008;9:69. (B) Oncomine boxed plot of *PTX3* expression levels between human non-metastatic and metastatic head and neck cancer in multiple datasets from ref. 1 (A) and ref. 2 (B). 1. Cromer A , et al. Oncogene 2004;23:2484-98. 2. Rickman DS, et al. Oncogene 2008;27:6607-22.

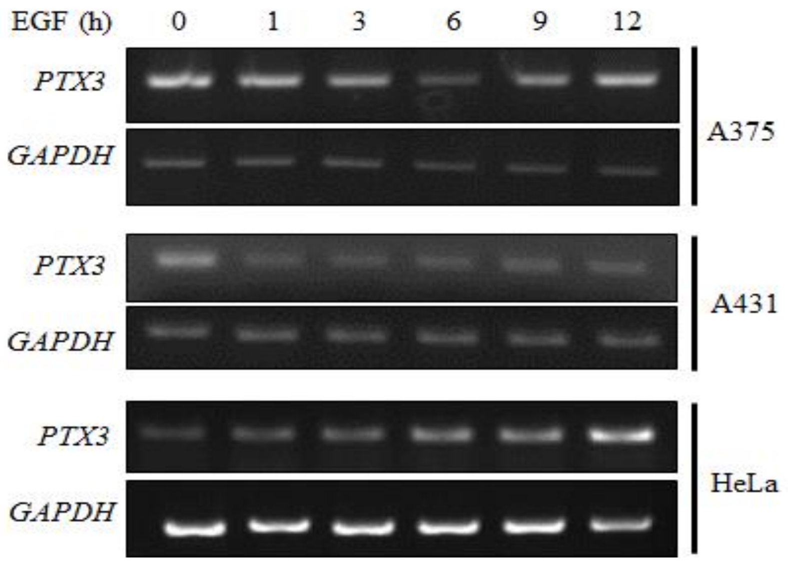

Supplementary Figure 2: Effect of EGF on PTX3 expression in various cancer cell lines. Cells were treated with 50 ng/ml EGF for a period of time as indicated. Expressions of *PTX3* and *GAPDH* mRNA were analyzed by an RT-PCR and examined in 2% agarose gels.

A

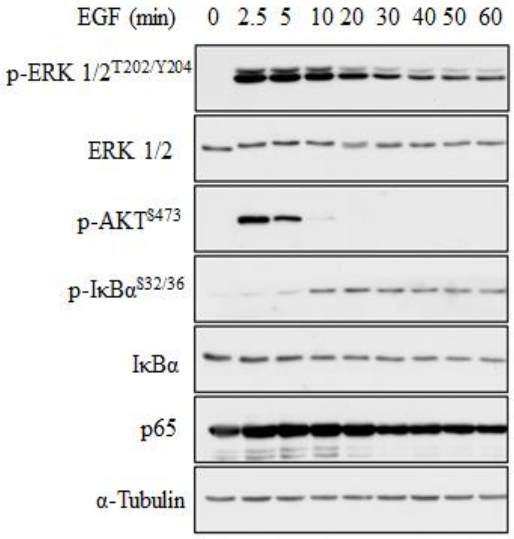

B

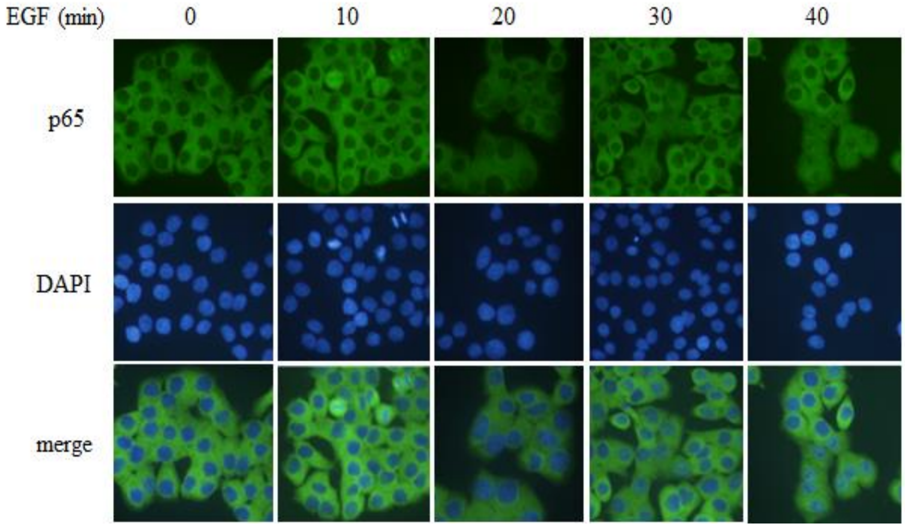

Supplementary Figure 3: Activation of ERK, Akt and NF-κB in EGF-treated cells. (A) KB cells were treated with 50 ng/ml EGF for a period of time as indicated. Lysates of cells were prepared and subjected to SDS-PAGE and analyzed by Western blotting with antibodies against phospho-ERK1/2, ERK1/2, phospho-Akt, phospho-IκBα, IκBα, p65, and α-tubulin. (B) KB cells were treated with 50 ng/ml EGF for a period of time as indicated. Anti-p65 antibodies and DAPI were used to respectively stain the nuclear translocation of NF-κB (p65) and DNA in an immunofluorescence analysis.

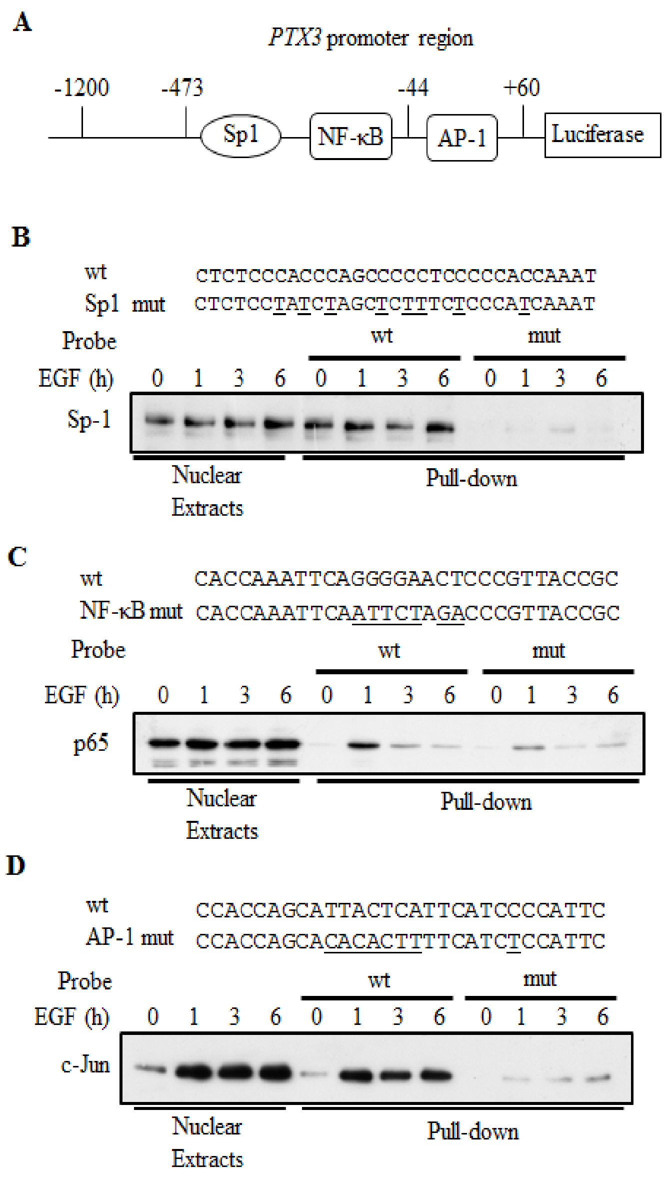

Supplementary Figure 4: Inhibition of Sp1, NF-Kb and c-Jun binding to PTX3 promoter in mutant binding sequences. (A) Constructs of the PTX3 promoter with Sp1-, NF-κB-, and AP1-binding sites bearing the luciferase gene are presented. Numbers indicate the number of base pairs upstream (-) and downstream (+) of the PTX3 translation start sites. (B-D) KB cells were treated with 50 ng/ml EGF for a period of time as indicated, nuclear extracts were prepared, and a DNA affinity precipitation assay (DAPA) was performed as described in “Materials and methods”. Binding of Sp1 (B), p65 (C), and c-Jun (D) to Sp1, NF-κB, and AP1 sites of the PTX3 promoter region (wt) or mutant probe (mut), respectively, was analyzed by Western blotting.

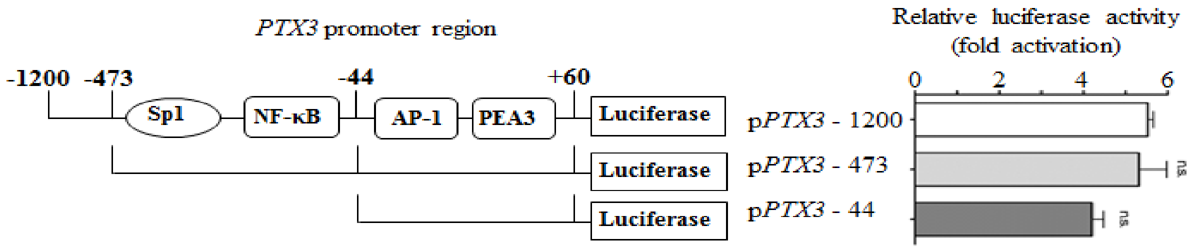

Supplementary Figure 5: Analysis of the response element of EGF-induced PTX3 promoter activity. Schematic representation of reporter constructs with the human PTX3 promoter shown on the left. Numbers indicate the number of base pairs upstream (-) and downstream (+) of the PTX3 translation start sites. Approximate locations of the putative Sp1-, NF-κB-, AP1-, and PEA3-binding motifs are indicated in the box. Multiples of induction of luciferase activity from these reporter constructs in KB cells treated with 50 ng/ml EGF for 6 h are shown on the right. Values are the mean ± S.E. of three determinations. n.s., no significant difference.

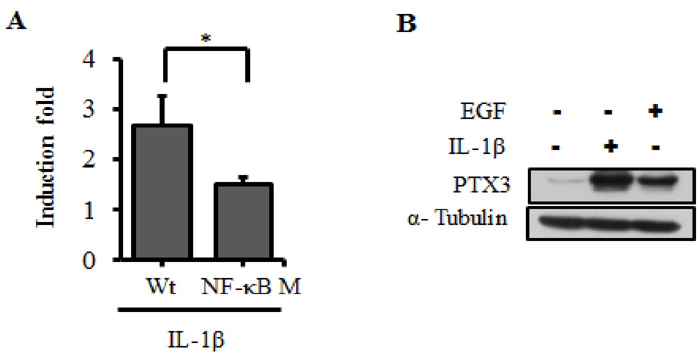

Supplementary Figure 6: The NF-κB-binding site located on the PTX3 promoter is essential for IL-1β-induced PTX3 expression. (A) KB cells were transfected with 0.5 µg wild-type (Wt) or NF-κB mutant (NF-κB M) of PTX3 promoter construct by lipofection and then treated with 5 ng/ml IL-1β for 6 h. Luciferase activities and protein concentrations were then determined and normalized. Values represent the mean ± S.E. of three determinations. (B) KB cells were treated with 50 ng/ml EGF or 5 ng/ml IL-1β for 6 h. Lysates of EGF-treated cells were prepared and subjected to SDS-PAGE and analyzed by Western blotting with antibodies against PTX3 and α-tubulin.

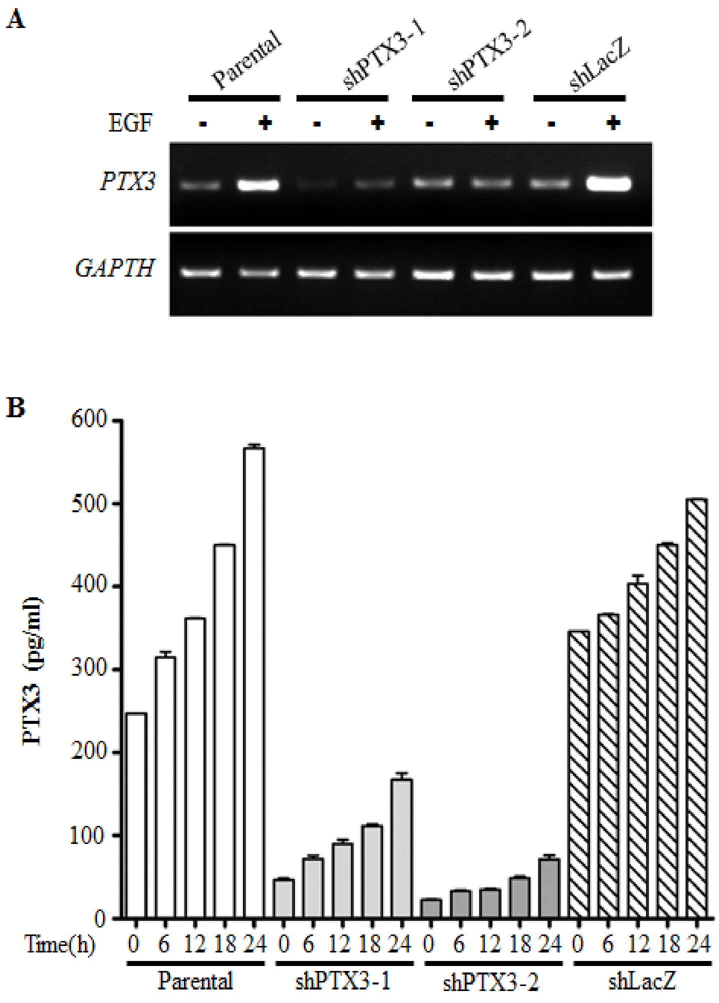

Supplementary Figure 7: Inhibition of EGF-induced PTX3 expression and secretion in shPTX3 cells. (A) KB cells were treated with 50 ng/ml EGF for 3 h. Expressions of *PTX3* and *GAPDH* mRNA were analyzed by an RT-PCR and examined in 2% agarose gels. shLacZ, negative control. (B) KB cells were treated with 50 ng/ml EGF for a period of time, and then conditioned media were collected to analyze PTX3 protein by an ELISA. Values are represented as the mean ± S.E. of three independent experiments. shLacZ, negative control.

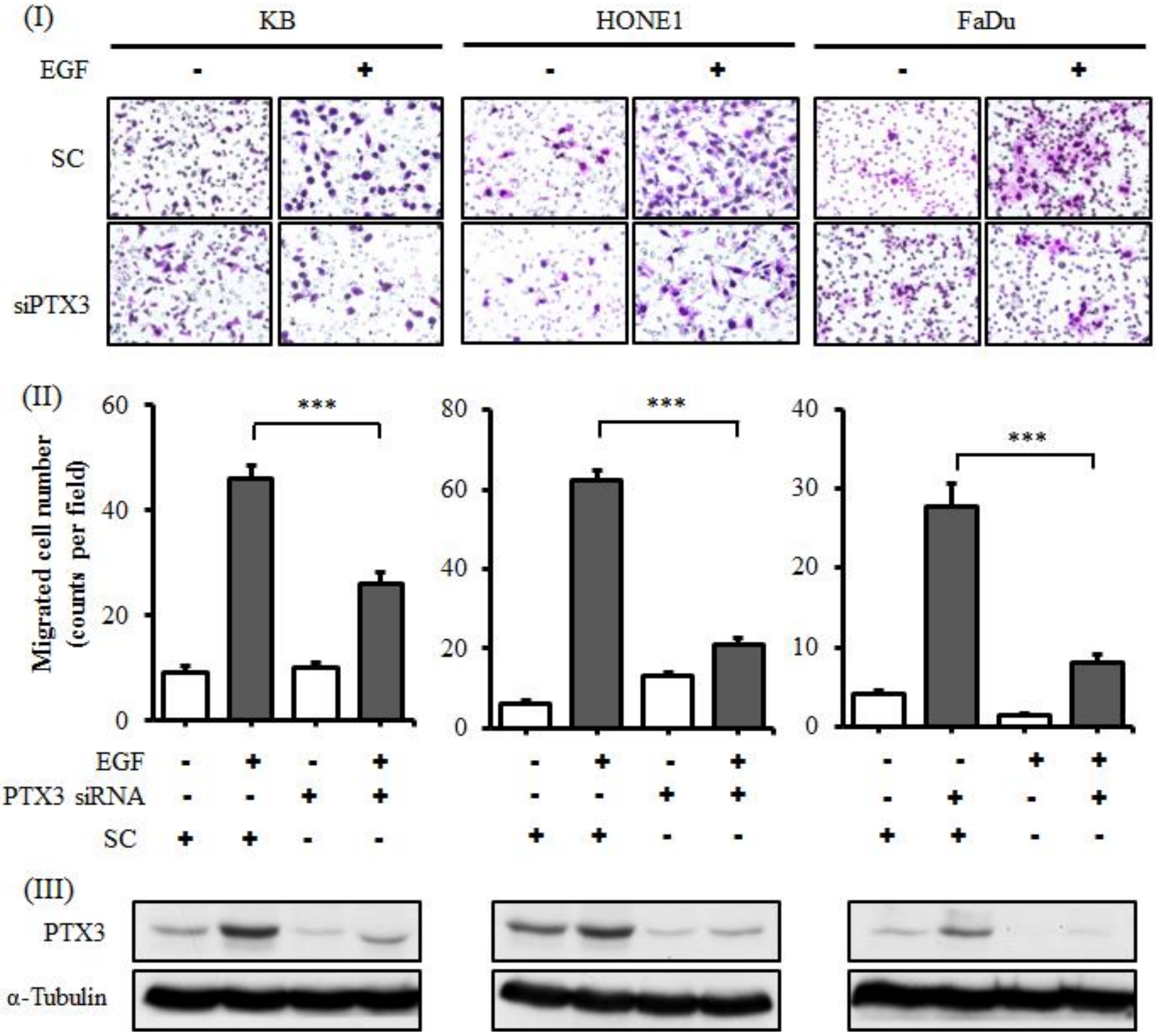

Supplementary Figure 8: Inhibition of EGF-induced HNSCC migration in PTX3-knockdown cells. Migratory properties of cells were analyzed by a transwell assay using Boyden chambers. Cells were transfected with 30 nM PTX3 siRNA oligonucleotides and scrambled oligonucleotides (SC) by lipofection. After treatment with 50 ng/ml EGF for 15 h, non-migratory cells were removed, and migratory cells were fixed and stained by a crystal violet procedure. The images of migration were examined using a microscope (I). Migrating cells were counted (II). Values are represented as the mean ± S.E. of three independent experiments. The expression of protein was analyzed by Western blotting with antibodies against PTX3 and α-tubulin (III).

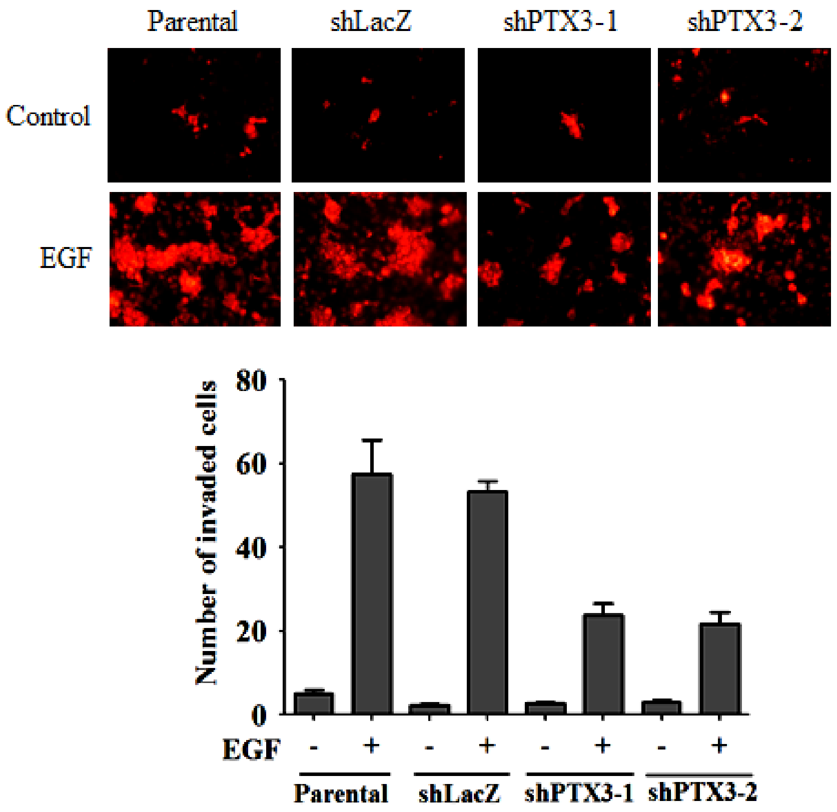

Supplementary Figure 9: Inhibition of EGF-induced cell invasion in PTX3-knockdown cells. Transendothelial invasion assay was performed as described in "Materials and methods". Parental and shPTX3 FaDu cells were treated with 50 ng/ml EGF for 6 h and stained with DiI, and then were loaded in the upper chamber of filter inserts. After incubation for 48 h, the non-invasive cells were removed. The invasive images were examined using a microscope (upper panel). The number of invasion cells was counted using three randomly chosen fields under the microscope from three independent experiments (lower panel). Error bars indicate SEM. shLacZ, negative control.

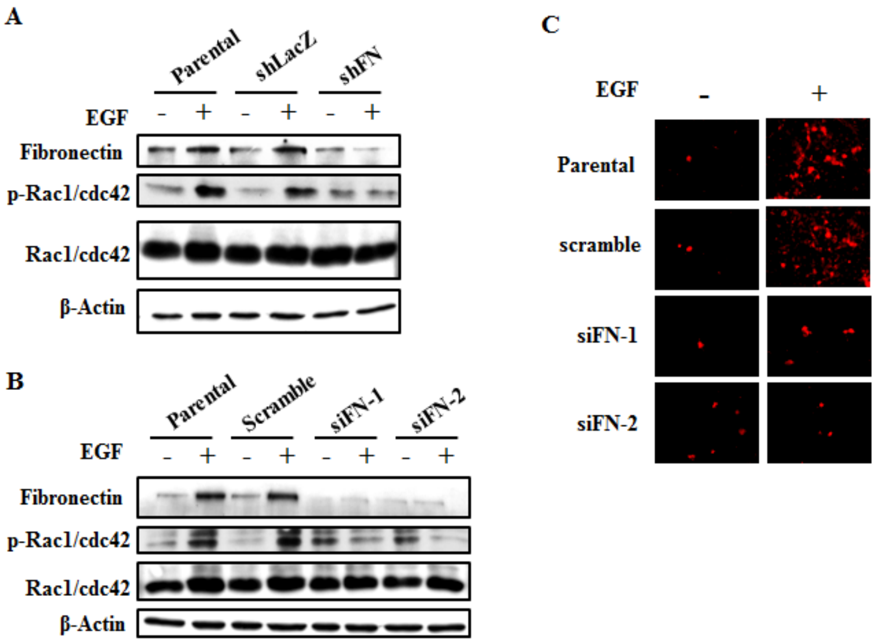

Supplementary Figure 10: The depletion of fibronectin inhibits epidermal growth factor (EGF)-induced phosphorylation of Rac1/cdc42 and cell invasion. (A) Parental and fibronectin knockdown (shFN) HONE1 cells were treated with 50 ng/ml EGF for 9 h. Cell lysates were prepared, subjected to SDS-PAGE and analyzed by western blotting with antibodies against fibronectin, Rac1/cdc42, phosphorylation of Rac1/cdc42 and β-actin. shLacZ, negative control. (B) HONE1 cells were transfected with 30 nM various fibronectin siRNA oligonucleotides (siFN-1 and siFN-2) and scrambled oligonucleotides (SC) by lipofection. After treatment with 50 ng/ml EGF for 9h, cell lysates were prepared, subjected to SDS-PAGE and analyzed by Western blotting with antibodies against fibronectin, Rac1/cdc42, phosphorylation of Rac1/cdc42 and β-actin. (C) Transendothelial invasion assay was performed as described in "Materials and methods". HONE1 cells were transfected with 30 nM various fibronectin siRNA oligonucleotides (siFN-1 and siFN-2) and scrambled oligonucleotides (SC) by

lipofection. After treatment with 50 ng/ml EGF for 6 h, cells were stained with DiI and then were loaded in the upper chamber of filter inserts. Images of invaded cells were captured under a microscope. Original magnification, ×200.
